# Draft genome sequence of a multidrug-resistant *Klebsiella pneumoniae* MTRKPMMI01 strain isolated from a diarrheic dairy cow in Bangladesh

**DOI:** 10.1128/mra.00773-26

**Published:** 2026-09-21

**Authors:** Md. Monirul Islam, Anindita Ash Prome, Sadia Afrin Punom, Md. Makshuder Rahman Zim, Md. Raisul Islam, S. M. Abu Sama Al Faruquee, Ram Narayan Yadav, Afsan Sarwer, Atikur Rahaman, Saifur Rahman, Md. Bahanur Rahman, Md. Tanvir Rahman

**Affiliations:** 1 Department of Microbiology and Hygiene, Faculty of Veterinary Science, Bangladesh Agricultural University 54492, Mymensingh, Bangladesh; Wellesley College, Wellesley, Massachusetts, USA

**Keywords:** whole-genome sequencing, *Klebsiella pneumoniae*, MDR, dairy cattle, Bangladesh

## Abstract

We report the draft genome sequence of *Klebsiella pneumoniae* strain MTRKPMMI01, isolated from a rectal swab of a diarrheic dairy cow from a dairy farm in Mymensingh city, with a total length of 5,239,585 bp, 3 plasmid replicons, 41 predicted antimicrobial resistance genes, and 93 predicted virulence factor genes.

## ANNOUNCEMENT

*Klebsiella pneumoniae* poses a growing threat to human and dairy cattle health (1). Dairy farms are potential sources of these zoonotic bacteria (2). Here, we present a draft genome sequence of multidrug-resistant *Klebsiella pneumoniae* MTRKPMMI01 strain isolated from rectal swabs of diarrhea-affected dairy cattle.

On 30 March 2026, a rectal swab was aseptically collected from a 3.5-year-old dairy cow with a 10-day history of diarrhea in Kewatkhali, Sadar, Mymensingh (24.7159°N, 90.4303°E). The swab was transferred immediately to 5 mL sterile nutrient broth and transported to the laboratory in an insulated ice box while maintaining a cold chain (approximately 4–8°C). The samples reached the laboratory within 2 h of collection. Following overnight incubation at 37°C, a loopful of the enriched broth culture was streaked onto MacConkey Agar (HiMedia, India) and incubated aerobically at 37°C overnight. A single lactose-fermenting (mucoid, pink) colony was subcultured twice to obtain a pure culture. Isolation and identification were performed based on colony morphology, Gram staining, and MALDI-TOF (3). The strain was identified as MDR through the Kirby-Bauer disk diffusion test (4). Bacterial isolate was then cultured in Luria-Bertani broth (BD, USA; catalog no. 244620), and DNA was extracted from the cultured broth using the DNeasy Blood & Tissue Kit (Qiagen, Germany; catalog no. 69504) according to the manufacturer’s instructions. Subsequently, the DNA library was prepared with the Illumina DNA Prep Reagent Kit (Illumina, USA; catalog no. 20060059), and sequencing was conducted on the Illumina NextSeq 2000 platform, generating paired-end reads with a 2 × 150 bp length. Raw Illumina paired-end reads were initially assessed for quality using FastQC v0.12.1 (5). Adapter sequences and low-quality bases were removed using fastp v0.23.4 (6) with default parameters, and the filtered high-quality reads were subsequently assembled *de novo* using SPAdes v13.13.1 (7). Genome annotation was performed in NCBI Prokaryotic Genome Annotation Pipeline v6.10 (8). Taxonomic classification and contamination (0.86%) were screened using Kraken2 v2.1.3 (9), and genome completeness (98.55%) was assessed with CheckM v1.2.5 (10). The sequence type was predicted using MLST v2.11 (https://github.com/tseemann/mlst), and virulence factor genes (VFGs) were identified using the virulence factor database (11) via ABRicate (https://github.com/tseemann/abricate). Moreover, the pathogenicity index, plasmids, antibiotic resistance genes (ARGs), bacteriocin gene clusters, prophages, and metabolic subsystems were predicted using PathogenFinder v2.0.0 (12), PlasmidFinder v2.1 (13), the comprehensive antibiotic resistance database with RGI main v6.0.2 (14), BAGEL4 (15), PHASTEST v1.0.1 (16), and RAST v2.0 (17), respectively. Unless otherwise noted, default parameters were used for all software. The genome characteristics of the *K. pneumoniae* MTRKPMMI01 strain are given in Table 1. This genome comprises 5,239,585 bp with a GC content of 57.5%. *De novo* assembly produced 94 contigs (>500 bp) with an N50 of 304,115 bp. Genome identity was confirmed by NCBI taxonomy check using average nucleotide identity (ANI) (18) with 99.15% identity to the *K. pneumoniae* reference type strain. The genome was identified as sequence type ST107 using seven-gene multilocus sequence typing (*gapA*, *infB*, *mdh*, *pgi*, *phoE*, *rpoB*, and *tonB*). The strain had a high predicted pathogenicity score of 0.909, matching 295 known pathogenic families, indicating substantial potential for virulence in both animals and humans.

**TABLE 1:** Genomic features of the *Klebsiella pneumoniae* strain MTRKPMMI01 isolated from a diarrheic dairy cow

Attributes	Values
BioProject accession	PRJNA1460333
GenBank accession	JBYAXA000000000
BioSample accession	SAMN57590771
SRA accession	SRR38331909
Assembled genome size (bp)	5,239,585
Guanine–cytosine (%)	57.5
Genome completeness (%)	98.55
Contamination (%)	0.86
Genome coverage	104.4×
N50 value (bp)	304,115
Predicted number of plasmid replicons	3 (Col440I, IncFII(K), IncR)
Number of contigs	94
Genes (total)	5,217
CDSs (total)	5,086
Genes (coding)	4,624
CDSs (with protein)	4,995
Genes (RNA)	131
rRNAs	8, 13, 15 (5S, 16S, 23S)
Complete rRNA	5, 5S
Partial rRNAs	3, 13, 15 (5S, 16S, 23S)
tRNAs	81
ncRNAs	14
Predicted number of ARGs	41
Predicted number of VFGs	93
Number of subsystems	380
Pathogenicity score	0.909
Matched with pathogenic families	295
ANI (%)	99.15
Reference genome (RefSeq)	GCF_057444835.1-RS_2026_05_14
Predicted number of intact prophages	1
Predicted number of bacteriocin clusters	2
Antimicrobial susceptibility test by disk diffusion methods	Resistant to ampicillin, tetracycline, ceftriaxone, cefotaxime, and fosfomycin; intermediate to amikacin, gentamicin, ciprofloxacin, meropenem, and imipenem; susceptible to chloramphenicol and levofloxacin

## Data Availability

All data and accession numbers are available in Table 1 and under the following accessions: GenBank, JBYAXA000000000; BioProject, PRJNA1460333; BioSample, SAMN57590771; and SRA, SRR38331909.

## References

[1] Yang Y, Higgins CH, Rehman I, Galvao KN, Brito IL, Bicalho ML, Song J, Wang H, Bicalho RC. 2019. Genomic diversity, virulence, and antimicrobial resistance of *Klebsiella pneumoniae* strains from cows and humans. Appl Environ Microbiol 85:e02654-18. doi:10.1128/AEM.02654-18 PMC6414388 30610074

[2] Holzhauer M, Wennink GJ. 2023. Zoonotic risks of pathogens from dairy cattle and their milk-borne transmission. J Dairy Res 90:325–331. doi:10.1017/S0022029923000730 38186208

[3] Patra JK, Das G, Das SK, Thatoi H. 2020. Isolation, culture, and biochemical characterization of microbes, p 83–133. *In* A practical guide to Environmental Biotechnology. Learning materials in biosciences. Springer, Singapore.

[4] Catalano A, Iacopetta D, Ceramella J, Scumaci D, Giuzio F, Saturnino C, Aquaro S, Rosano C, Sinicropi MS. 2022. Multidrug resistance (MDR): a widespread phenomenon in pharmacological therapies. Molecules 27:616. doi:10.3390/molecules27030616 PMC8839222 35163878

[5] Andrews S. 2010. FastQC: a quality control tool for high-throughput sequence data. Available from: http://www.bioinformatics.babraham.ac.uk/projects/fastqc

[6] Chen S, Zhou Y, Chen Y, Gu J. 2018. Fastp: an ultra-fast all-in-one FASTQ preprocessor. Bioinformatics 34:i884–i890. doi:10.1093/bioinformatics/bty560 PMC6129281 30423086

[7] Bankevich A, Nurk S, Antipov D, Gurevich AA, Dvorkin M, Kulikov AS, Lesin VM, Nikolenko SI, Pham S, Prjibelski AD, Pyshkin AV, Sirotkin AV, Vyahhi N, Tesler G, Alekseyev MA, Pevzner PA. 2012. SPAdes: a new genome assembly algorithm and its applications to single-cell sequencing. J Comput Biol 19:455–477. doi:10.1089/cmb.2012.0021 PMC3342519 22506599

[8] Tatusova T, DiCuccio M, Badretdin A, Chetvernin V, Nawrocki EP, Zaslavsky L, Lomsadze A, Pruitt KD, Borodovsky M, Ostell J. 2016. NCBI prokaryotic genome annotation pipeline. Nucleic Acids Res 44:6614–6624. doi:10.1093/nar/gkw569 PMC5001611 27342282

[9] Wood DE, Lu J, Langmead B. 2019. Improved metagenomic analysis with Kraken 2. Genome Res 25:1043–1055. doi:10.1101/gr.186072.114 PMC6883579 31779668

[10] Parks DH, Imelfort M, Skennerton CT, Hugenholtz P, Tyson GW. 2015. CheckM: assessing the quality of microbial genomes recovered from isolates, single cells, and metagenomes. Genome Res 25:1043–1055. doi:10.1101/gr.186072.114 PMC4484387 25977477

[11] Chen L, Zheng D, Liu B, Yang J, Jin Q. 2016. VFDB 2016: hierarchical and refined dataset for big data analysis–10 years on. Nucleic Acids Res 44:D694–D697. doi:10.1093/nar/gkv1239 PMC4702877 26578559

[12] Ferrer Florensa A, Almagro Armenteros JJ, Kaas RS, Conradsen Clausen PTL, Nielsen H, Rost B, Aarestrup FM. 2025. Whole-genome prediction of bacterial pathogenic capacity on novel bacteria using protein language models, with PathogenFinder2. Bioinformatics. doi:10.1101/2025.04.12.648497 PMC13218381 41863303

[13] Carattoli A, Zankari E, García-Fernández A, Voldby Larsen M, Lund O, Villa L, Møller Aarestrup F, Hasman H. 2014. *In silico* detection and typing of plasmids using PlasmidFinder and plasmid multilocus sequence typing. Antimicrob Agents Chemother 58:3895–3903. doi:10.1128/AAC.02412-14 PMC4068535 24777092

[14] Alcock BP, Huynh W, Chalil R, Smith KW, Raphenya AR, Wlodarski MA, Edalatmand A, Petkau A, Syed SA, Tsang KK, et al.. 2023. CARD 2023: expanded curation, support for machine learning, and resistome prediction at the Comprehensive Antibiotic Resistance Database. Nucleic Acids Res 51:D690–D699. doi:10.1093/nar/gkac920 PMC9825576 36263822

[15] van Heel AJ, de Jong A, Song C, Viel JH, Kok J, Kuipers OP. 2018. BAGEL4: a user-friendly web server to thoroughly mine RiPPs and bacteriocins. Nucleic Acids Res 46:W278–W281. doi:10.1093/nar/gky383 PMC6030817 29788290

[16] Wishart DS, Han S, Saha S, Oler E, Peters H, Grant JR, Stothard P, Gautam V. 2023. PHASTEST: faster than PHASTER, better than PHAST. Nucleic Acids Res 51:W443–W450. doi:10.1093/nar/gkad382 PMC10320120 37194694

[17] Aziz RK, Bartels D, Best AA, DeJongh M, Disz T, Edwards RA, Formsma K, Gerdes S, Glass EM, Kubal M, et al.. 2008. The RAST Server: rapid annotations using subsystems technology. BMC Genomics 9:1–15. doi:10.1186/1471-2164-9-75 PMC2265698 18261238

[18] Jain C, Rodriguez-R LM, Phillippy AM, Konstantinidis KT, Aluru S. 2018. High throughput ANI analysis of 90K prokaryotic genomes reveals clear species boundaries. Nat Commun 9:5114. doi:10.1038/s41467-018-07641-9 PMC6269478 30504855

